# 

**Published:** 1872-11

**Authors:** 


### Books and Pamphlets Received.

A Practical Treatise on Urinary and Renal Diseases, including Urinary Deposits. By William Roberts, M. D., etc. Second American from the Second Revised London Edition. Philadelphia: Henry C. Lea, 1872. Buffalo: T. Butler & Son.

Transactions of the American Medical Association. Vol. XXIII. Philadelphia : Printed for the Association. Collins, Printer, 1872. Buffalo : T. Butler & Son.

Lessons in Physical Diagnosis. By Alfred Loomis, M. D., etc. Third Revised and Enlarged Edition. New York : Wm. Wood & Co., 1872. Buffalo : H. H. Otis & Co.

General and Differential Diagnosis of Ovarian Tumors, with special reference to the Operation of Ovariotomy ; and Occasional Pathological and Therapeutical Considerations. By Washington L. Atlee, M. D. Philadelphia : J. B. Lippincott & Co., 1873. Buffalo : H. H. Otis & Co.

A Hand-Book of Compound Medicines ; or, the Prescriber's and Dispenser's Vade-Mecum. By Arnold J. Cooley. Philadelphia : J. B. Lippincott & Co., 1873. Buffalo : H. H. Otis & Co.

Practical Lessons in the Nature and Treatment of the Affections Produced by the Contagious Diseases ; with an Account of the Primary Syphilitic Poison and of its Communicability. Philadelphia : J. B. Lippincott & Co., 1873. Buffalo : H. H. Otis & Co.

The Physicians' Visiting List for 1873. Philadelphia : Lindsay & Blakiston. Sold by all Booksellers and Druggists.

Transactions of the Sixth Annual Meeting of the Medical Association of the State of Missouri, held in St. Joseph, Mo., April, 1872.

What Physiological Value Has Phosphorus as an Organismal Element ? An Essay, to which was awarded the prize of the American Medical Association for 1872. By Samuel R. Percy, M. D., etc.

Half-Hour Recreations in Popular Science. Dana Estes, Editor. No. 5. Nebulæ, Comets, Meteoric Showers, and the revelations of the Spectroscope concerning them. By Prof. H. Schellen and others. Coral and Coral Islands. By Prof. J. D. Dana, of Yale. Boston : Estes & Lauriat, 1872. Buffalo : Martin Taylor. H. H. Otis & Co.

Medical and General Science as Vindicators of the Mosaic Record. By E. S. Gaillard, M. D., etc. From October No. Richmond and Louisville Medical Journal.

Clinical Analysis of the Inflammatory Affections of the Inner Ear. By H. Knapp, Surgeon to New York Opthalmic and Aural Institute. New York : Wm. Wood & Co,, 1871.

Recent Researches in Electro-Therapeutics. By George M. Beard, M. D. New York : D. Appleton & Co. From October No. New York Medical Journal.

Report on the Structure of the White Blood Corpuscle. By Jos. G. Richardson, M. D. From Transactions of the American Medical Association.

On a New Method for Extraction of Cataract. By R. Liebreich, Opthalmic Surgeon to, and Lecturer on Ophthalmology at, St. Thomas's Hospital. Philadelphia : Claxton, Remsen & Haffelfinger. 1873.

Vick's Floral Guide for 1873. Issued by James Vick, Rochester, N. Y.

